# Decoding the role of cytochrome c in metabolism of human spermatozoa by Raman imaging

**DOI:** 10.3389/fcell.2022.983993

**Published:** 2022-11-25

**Authors:** Halina Abramczyk, Bogna Sobkiewicz, Renata Walczak-Jędrzejowska, Katarzyna Marchlewska, Jakub Surmacki

**Affiliations:** ^1^ Lodz University of Technology, Institute of Applied Radiation Chemistry, Laboratory of Laser Molecular Spectroscopy, Lodz, Poland; ^2^ Fertility Clinic Salve Medica, Lodz, Poland; ^3^ Department of Andrology and Reproductive Endocrinology, Medical University of Lodz, Lodz, Poland

**Keywords:** human spermatozoa, semen analysis, cytochrome c, mitochondria, Raman spectroscopy and imaging

## Abstract

The normal functioning of sperm cells requires cytochrome c in the redox balanced forms: reduced and oxidized. The oxidized form of cytochrome c is localized in the mitochondrial intermembrane space and is a part of the electron transport chain. This ensures that electron shuttling between the complex III, cytochrome c, and complex IV can occur leading to controlled effective oxidative phosphorylation (respiration) and ATP production needed for most steps in spermatozoal maturation, motility, hyperactivation and fertilization. We studied the biochemical composition of specific organelles in sperm cells by Raman imaging. The structures of the head consisting of the nucleus and acrosome, the midpiece representing mitochondria, and the tail characterized by the sperm axoneme surrounded by outer dense fiber and covered by the membrane were measured. Metabolic biochemical analysis of mitochondria, head and tail of sperm cells, and seminal plasma by using Raman imaging combined with chemometric classification method of Cluster Analysis has been obtained. Our results show that cytochrome c, which is a key protein that is needed to maintain life (respiration) and cell death (apoptosis), is located in sperm mitochondria in the oxidized or reduced form of the heme group. This work demonstrated that an application of Raman micro-spectroscopy can be extended to monitoring the redox state of mitochondrial cytochrome c in sperm cells.

## 1 Introduction

The combination of female and male reproductive cells makes it possible to create a new life. It is a complicated and multi-stage process that begins from oogenesis and spermatogenesis up to fertilization. Male or female infertility depends on many factors. A routine semen analysis identifies male infertility (Esteves et al., 2011), but it is difficult to determine a threshold to distinguish the fertile from infertile men based on ejaculate results (Schjenken and Robertson, 2015). Human semen quality is evaluated by semen volume, sperm concentration, motility, vitality and morphology (Cooper et al., 2010; WHO, 2021). The main parameter for classification of idiopathic infertile men was low sperm motility [<42% motile spermatozoa; >16 million spermatozoa/ml; > 4% normal forms (WHO, 2021)]. A high-throughput, fast analysis of proteins, metabolites, hormones, organic acids, lipids, nucleosides, minerals, and vitamins can be used as a screening tool to provide a semen sample classification as fertile/infertile or healthy/unhealthy (Ellis et al., 2007). Unfortunately, in spite of progress in the identification of the fertile/infertile or healthy/unhealthy samples in the recent decades, our understanding of molecular mechanisms occurring in the male reproductive cells did not make significant progress.

Many of the papers focus on oxidative stress on the pathophysiology of mitochondrial dysfunction in human spermatozoa. Susceptible to oxidative damage sperm mtDNA can affect sperm function leading to infertility *via* increased mtDNA copy number and reduced mtDNA integrity (Durairajanayagam et al., 2021). However, male fertility depends on many other factors, one of them being the normal functionality of cytochrome c localized in the mitochondrial intermembrane space being a part of the electron transport chain (ETC). Cytochrome c is a key protein that is needed to maintain life (respiration) and cell death (apoptosis). The dual-function of cytochrome c comes from its capability to act as a mitochondrial redox carrier that transfers electrons between the membrane-embedded complexes III and IV and to serve as a cytoplasmic apoptosis-triggering agent, activating the caspase cascade. When an oxidized form of cytochrome c is released into the cytosol can induce caspase activation *via* the apoptosome, while the reduced form cannot (Riedl and Salvesen, 2007; Ripple et al., 2010). Activated caspases cleave cellular proteins that result in the cellular hallmarks of apoptosis. In its role in the ETC, cytochrome c shuttles electrons between the cytochrome c_1_ center of the bc_1_ complex (complex III), and the CuA center of cytochrome c oxidase (CytOx) (Kokhan et al., 2010). The movement of electrons along the ETC is coupled to proton extrusion from the matrix to the mitochondrial intermembrane space by complex I, the bc_1_ complex and CytOx creating a chemiosmotic potential for protons across the inner membrane. The energy released when protons are returned to the matrix is used by the ATP synthase to generate ATP from ADP and inorganic phosphate (Ripple et al., 2010). However, the precise roles of cytochrome c in mitochondria, cytoplasm and extracellular matrix under normal and pathological conditions of reproductive processes and are not completely understood. To date, the significance of mitochondrial dysfunctionality has not been studied in reproductive cells to the best of our knowledge. Some researchers identified cytochrome c (Meister et al., 2010) in human sperm at ∼751 cm^−1^, which was assigned to mitochondrial cytochrome c (Adar and Erecinska, 1978; Takahashi and Ogura, 2002; Berezhna et al., 2003; Onogi et al., 2010; Okada et al., 2012). However, Mallidis et al, (2011) and Amaral et al, (2018) questioned this assignment, reporting this peak in the spectrum of other regions in the human sperm.

The pioneer of the field of sperm analysis was Kubasek et al. 1986) who first applied Raman spectroscopy to the study of salmon sperm DNA (Kubasek et al., 1986). The following decades did not make significant progress, but recently the situation changed due to the update of Raman imaging and the improvement of chemometric methodology. Raman spectroscopy and imaging have been applied to the scientific research and clinical application of sperm analysis by more and more researchers (Li et al., 2014; Liu et al., 2014; Amaral et al., 2018; Costa et al., 2018; Mallidis and Wistuba, 2018; De Angelis et al., 2019; Fikiet and Lednev, 2019; Pourasil and Gilany, 2021).

In this paper, we will concentrate on the role of cytochrome c in mitochondria, which may be the key molecule deciding on the life and death of human male reproductive cells. We used Raman spectroscopy and imaging to monitor changes in the redox state of the mitochondrial cytochromes in sperm cells.

## 2 Materials and methods

### 2.1 Ethical approval

Written informed consent to participate in this study was provided by the semen donors attending the Infertility Clinic Salve Medica, Łódź, Poland and the studies were reviewed and approved by the Department of Clinical Trials in Salve Medica and the institutional Bioethical Committee at the Medical University of Lodz, Poland (RNN/253/22/KE). All procedures were conducted in accordance with the guidelines of the Declaration of Helsinki (2013).

### 2.2 Semen samples

Human semen samples were obtained from 11 donors. Samples were collected after 2–7 days of sexual abstinence. After liquefaction basic examination according to WHO 2021 [WHO 2021 p.164 (WHO, 2021)] recommendation was performed for diagnostic purpose. All analyzed samples have parameters above the lower reference limits. After examination, a residual material was prepared for further a confocal Raman micro-spectroscopy analysis.

### 2.3 Confocal Raman micro-spectroscopy

10 μl of the native semen samples from 11 donors were smeared on a CaF_2_ Raman grade window slides and air-dried before Raman evaluation. For each donors Raman imaging has been performed for at least 3 sperm cells, and additional sperm cells were 10 points line scanned in the three positions (head, midpiece and tail) it the fingerprint (400–1800 cm^−1^) and high-frequency (2500–3100 cm^−1^) regions.

Raman spectra and images were recorded using a confocal Raman microscope (WITec (alpha 300 RSA+), Ulm, Germany) in the Laboratory of Laser Molecular Spectroscopy, Lodz University of Technology, Poland. The Raman microscope consisted of an Olympus microscope (Olympus Düsseldorf, Germany) a UHTS (Ultra-High-Throughput Screening) monochromator (WITec, Ulm, Germany) using a 1200 grooves/mm diffraction grating. These settings provide a spectral resolution of 2.22 cm^−1^/pixel, showing an optimal signal-to-noise ratio. For all measurements, a 100x objective (NA = 0.9) with a working distance of 0.21 mm was used and they were performed within a spectral range of 600–1800 cm^−1^and a thermoelectrically cooled CCD camera ANDOR Newton DU970N-UVB-353 (EMCCD (Electron Multiplying Charge Coupled Device, Andor Technology, Belfast, Northern Ireland) chip with 1600 × 200 pixel format, 16 µm dimension each) at −60°C with full vertical binning. The excitation laser at 532 nm was focused on the sample to the laser spot of 1 µm and was coupled to the microscope *via* an optical fiber with a diameter of 50 µm. The average laser excitation power was 10 mW, and the collection time was 0.5 for Raman images. Raman images were recorded with a spatial resolution of 1 × 1 µm. A typical Raman map of a sperm cell consists of 1680 Raman spectra (map size 35 × 12 μm). The Raman spectrometer was calibrated every day prior to the measurements using a silica plate with a maximum peak at 520.7 cm^−1^. We collected the Raman data using the electron multiplying mode of the CCD camera, which guarantees a better signal-to-noise ratio and allowed us to reduce integration time to 0.5 s. Longer acquisition time is time-consuming and extends the measurement time of the Raman maps or the line scans effecting the statistics. We carefully checked the possible thermal effect on the sample using 10 mW and evidently, we did not observe the sample deterioration.

### 2.4 Data analysis

The obtained Raman data were analyzed by Cluster Analysis using the Project Plus (WITec GmbH, Germany), Origin 2016 (Origin Lab, United States) and Principal Component Analysis (PCA) was performed using MATLAB (MathWorks, United States) with PLS-Toolbox (Eigenvector Research Inc., United States). Raman spectra of sperm samples were background subtracted (mode: polynominal). Only in the PCA analysis, spectra were normalized (preprocessing PCA mode: SNV normalization). PCA analysis is a dimension reduction analysis that allows the identification of patterns in high dimensional data, expressing the data in such a way as to highlight their similarities and differences.

### 2.5 Cytochrome c

Cytochrome c from the equine heart (C7752, Sigma-Aldrich) was used without additional purification. Cytochrome c solutions with concentration in the range of 0–0.46 mM was prepared in phosphate buffer (PBS, 10010023, Gibco). Ferrous cytochrome c was prepared by adding 10-fold excess NaBH_4_ (as a reductor).

## 3 Results

### 3.1 The biochemical composition of specific organelles in sperm cells by Raman imaging

**FIGURE 1:**
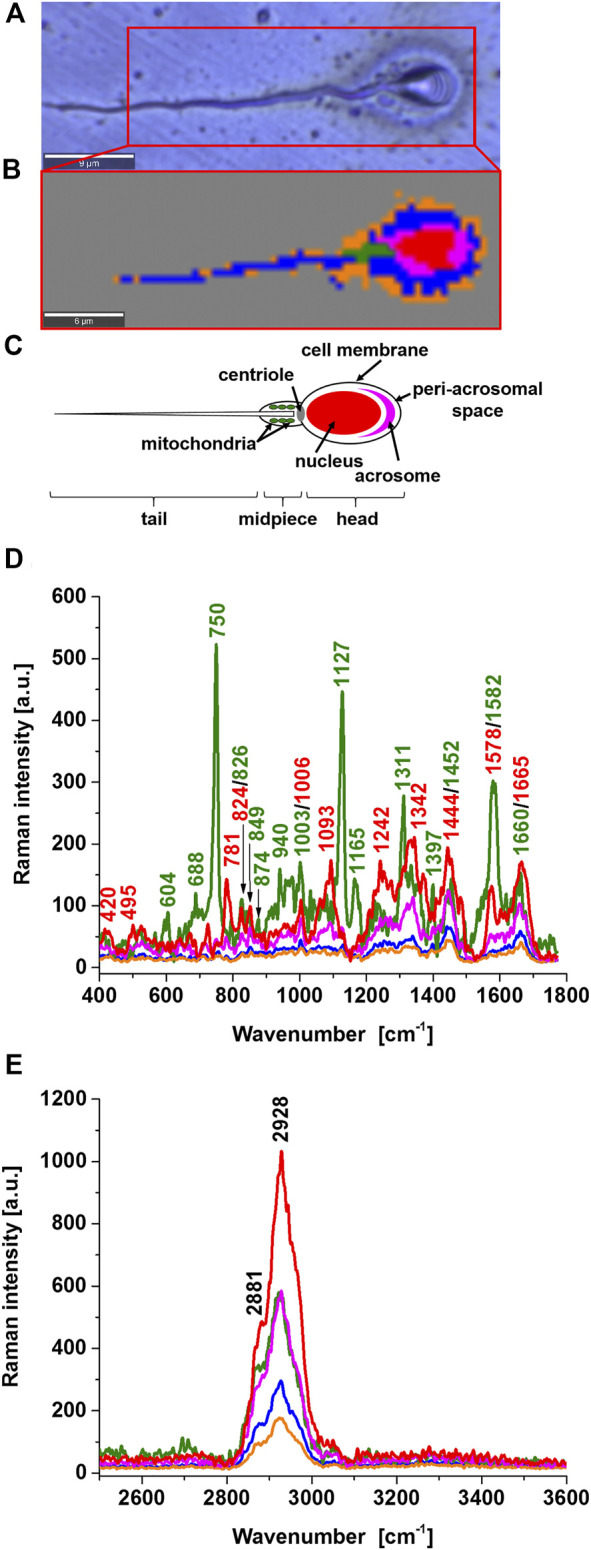
Microscope image **(A)**, Raman image **(B)**, diagram of a typical human sperm cell **(C)**, Raman spectra of a typical sperm cell regions: nucleus (red), mitochondrion (green), acrosome (magenta), axsoneme and membrane cell (blue), seminal plasma attached to the surface of the sperm cell (orange) in the fingerprint region **(D)** and high-frequency region **(E)**.

Figure 1 shows a microscopy image and a Raman image of a typical sperm cell. The Raman image was obtained from a collection of spatially resolved Raman spectra by raster-scanning the focused laser beam over the sample, *via* a high-resolution microscope stage. The resulting spectral data were converted into Raman spectral images improved images with the statistical methods of cluster analysis. The number of clusters in Figure 1B was 6 and represent the main sperm regions: the head consisting of the nucleus (red colour), acrosome (magenta), the midpiece representing mitochondria (green), and the tail characterized by the sperm axoneme (blue) that is surrounded by outer dense fiber covered by the membrane (orange) and area outside of the cell (dark grey). These specific structures have their characteristic Raman spectra presented in Figure 1D that provide information on the biochemical composition of specific organelles. This feature is the main advantage over microscopy image in Figure 1A and many other methods such as gas chromatography, mass spectrometry, nuclear magnetic resonance, and fluorescence. Until now, no technology has proven effective for detecting cytochrome c concentration in specific cell organelles. Therefore, existing analytical technologies cannot detect the full extent of cytochrome c localization inside and outside specific organelles. In Raman imaging, we do not need to disrupt cells to break open the cells and release the cellular structures to learn about their biochemical composition.

Comparing the sperm regions in Figure 1D one can observe the zones of different intensities associated with a the concentration of biochemical components and/or Resonance Raman enhancement. The Resonance Raman enhancement can occur only for the chemical components that have electron absorption in the region of the laser excitation (532 nm). Among the chemical components that fulfill the condition of resonance are carotenoids and heme proteins. Figure 1D shows the strongest Raman signal for the compound (green) characterized by the peaks 750, 1127, 1311, 1399, and 1582 cm^−1^. All of them perfectly correspond to the Raman vibrations of cytochrome c (Strekas and Spiro, 1972; Hu et al., 1993; Abramczyk et al., 2022). Carotenoids were not found.

Cytochrome c a heme protein has the electronic transition resonance for the Q absorption band with the used laser excitation at 532 nm. The peaks of cytochrome c in the Raman spectrum in Figure 1D are not observed in other regions in the human sperm. The assignment of cytochrome c Raman bands is in accordance with the assignments proposed by Adar and Erecinska, (1978), Takahashi and Ogura, (2002), Berezhna et al, (2003), Onogi et al, (2010) and in contrast to the assignments of Mallidis et al, (2011) and Amaral et al, (2018). The peak at 1578 cm^−1^, close to the band 1582 cm^−1^ is observed in the nucleus (red) but this band represents nucleic acids of nucleus DNA, not cytochrome c. Therefore, the midpiece corresponding to mitochondria is characterized by cytochrome c 750, 1127, 1311, 1399, and 1582 cm^−1^. The other bands of the mitochondria at 608, 688, 826, 849, 874, 940, 1003, 1093 1165, 1242, 1342, 1399, 1660 cm^−1^ represent vibrations of other proteins and lipids (e.g. 1003 cm^−1^ phenylalanine (symmetric ring breathing mode), 1093 cm^−1^symmetric PO^2−^ stretching vibration of the mtDNA backbone; 1660 cm^−1^amide I/C=C vibrations) (Huser et al., 2009).

It is important to emphasize that the band observed in the midpiece region at 1452 cm^−1^, which does not represent cytochrome c, is very important for the functioning of cytochrome c in the electron transfer chain. The band at 1452 cm^−1^ represents cardiolipin and does not overlap with the C-H deformation bands of saturated lipids at 1444 cm^−1^. Cytochrome c is mostly protonated meaning that most cytochrome c bonds *via* electrostatic bonds to acidic phospholipids, particularly cardiolipin. Cardiolipin-bound cytochrome c, probably does not participate in electron shuttling of the respiratory chain (Kagan et al., 2004). It indicates that the process of oxidative phosphorylation (respiration) becomes less effective in cancer cells (known as the Warburg effect). On the other hand, the reduced form of cytochrome c (Fe^2+^) cannot induce caspase activation and the process of apoptosis in cancerous cells becomes less efficient (Brown and Borutaite, 2008).

The sperm head consists of the nucleus (red color), surrounded by acrosome (magenta), peri-acrosomal space and cell membrane (blue), and seminal plasma attached to the surface of the sperm cell (orange) as shown in Figure 1B. The acrosome a cap-shaped area surrounding the nucleus in front of the sperm head plays a key role in fertilizing by producing hydrolytic enzymes that enable it to enter the egg cell. The nucleus is characterized by the bands at 781, 825, 1093, 1242, 1342, 1444, 1578, and 1665 cm^−1^ representing U, T, C (ring breathing modes in the DNA/RNA bases) (781 cm^−1^); phosphodiester,– O–P–O stretch DNA/RNA–ring breathing tyrosine (825 cm^−1^); symmetric PO^2−^ stretching vibration of the DNA backbone (1093 cm^−1^); Amide III and CH_2_ wagging vibrations from glycine backbone and proline side chain,—A, G (ring breathing modes in the DNA bases) (1242 cm^−1^); C–H deformation (protein) (1342 cm^−1^),CH_2_ and CH_3_ deformations (antisymmetric methyl and methylene deformations, peptide side chains, phospholipids),—CH_2_ bending mode of proteins and lipids,—CH_2_CH_3_ deformation,—C–H vibration (proteins and lipids),– CH_2_ bending mode of DNA, CH_2_ deformation (thymine); C = C bending mode of guanine (1444 cm^−1^); nucleic acid DNA modes: G, A ring breathing modes (1578 cm^−1^), and A, T, G, C (ring breathing modes of the DNA/RNA bases) (1665 cm^−1^).

The acrosome region is characterized by the bands characteristic for proteins (at 1665 cm^−1^ - Amide I; 1242cm^−1^—Amide III; 1444cm^−1^—C-H bending and deformation bands; 730cm^−1^—C–S, CH_2_ rocking–C–C stretching, proline) and DNA/RNA bases [at 781 cm^−1^—U, T, C (ring breathing modes in the DNA/RNA bases, backbone O–P–O)]. Both the nucleus and acrosome demonstrate the absence of peaks that are specifically assigned to mitochondrial cytochrome c. The peri-acrosomal space and cell membrane are characterized by the bands characteristic for proteins and lipids.

The region of the tail consists of the axoneme, outer dense fibers and membrane (represented by blue color in Figure 1B) and the seminal plasma attached to the surface of the sperm cell (orange color).

### 3.2 The redox balance of cytochrome in the midpiece region of mitochondria

Now is just the beginning of our understanding of metabolic factors involved in the development of sperm quality, but it has become evident from our results that sperm cells show many differences in the redox status of cytochrome c in mitochondria.

Now we will concentrate on the redox balance of cytochrome in the midpiece region of the sperm cell. This midpiece region contains mitochondria. Optimal mitochondrial activity is suggested to be a key factor (Breitbart, 2003; Jha et al., 2003; Bailey, 2010; Sakkas and Alvarez, 2010; Gupta and Bhandari, 2011; Aitken, 2017; Dutta et al., 2019; Ribeiro et al., 2021; Podolak et al., 2022) for semen quality and human sperm function in fertilization. However, the precise role of mitochondria in spermatozoa remains to be fully explored.

**FIGURE 2:**
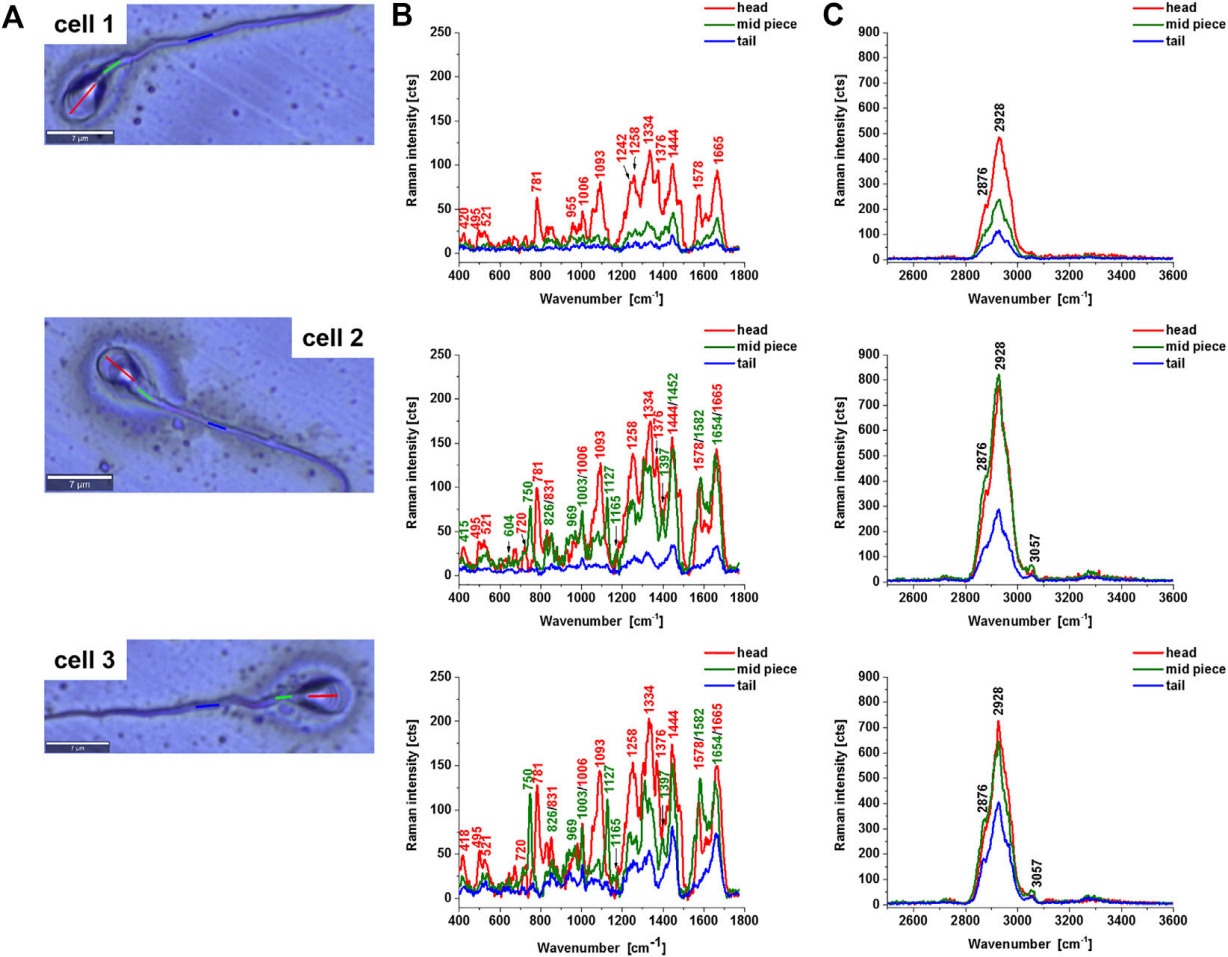
Microscope images of a human typical sperm cells **(A)**, the average Raman spectra of a typical sperm cell regions: head (red), midpiece (green), axsoneme and membrane cell (blue), in the fingerprint region **(B)** and high-frequency region **(C)**. Experimental condition: excitation 532 nm, laser power 10 mW, integration time 0.5 s, number of Raman spectra of each line n = 10.

Figure 2 shows Raman average spectra from 10 spectra from the sperm cell regions: head, midpiece tail (including the axsoneme) for three different sperm cells. In the head, we observe a nucleus that shows the same vibrational features as discussed above. Now, we will focus on the features of cytochrome c in mitochondria localized in the midpiece region.

First, one can see from Figure 2B that 1) the cytochrome c Raman bands intensities become lower than the Raman intensities of the nucleus for a cell 1 compared with the cells 2 and 3, 2) the ratio 1582/1665 represents the ratio of the Raman signals of cytochrome c at 1582 cm^−1^ and proteins and lipids at 1665 cm^−1^, changes significantly from 0.25 at Figure 2B (cell 1), 0.81 (Figure 2B cell2), 1.03 (Figure 2B cell 3). This ratio gives information about the concentration of cytochrome c to the total amount of proteins and lipids inside the sperm cell. We have focused on a fingerprint spectral range because in the fingerprint we observed the most characteristic spectra of cytochrome c. The high-frequency region provides information on the other proteins and lipids (Table 1).

**TABLE 1:** Raman band assignments from the head, midpiece and tail of sperm cell.

Raman band (cm^−1^)	Head	Midpiece	Tail	Assignment
608	+	+	+	C–C–C ring deformation
688		+		Ring deformation
730				C–S, CH_2_ rocking—C–C stretching, proline
∼748–750		+		Cytochrome c
781	+			DNA/RNA bases (uracil, thymine, cytosine), ring breathing modes
∼825–826	+	+		phosphodiester,– O–P–O stretch DNA/RNA– ring breathing tyrosine
849	+	+		Proline, hydroxyproline, tyrosine ring breathing mode
874	+	+		Carbohydrate, C–O–C skeletal mode
940		+		Carbohydrates, skeletal mode
1003		+	+	Proteins, phenylalanine (symmetric ring breathing mode)
∼1093	+	+		DNA, symmetric PO^2−^ stretching vibration of the mtDNA backbone
1127		+		Cytochrome c
1165		+		Proteins,—C–H in-plane bending mode of tyrosine,—(CH) Phenylalanine
∼1242–1258	+	+	+	Amide III and CH_2_ wagging vibrations from glycine backbone and proline side chain; adenine, guanine (ring breathing modes in the DNA bases)
∼1304–1311		+		Cytochrome c
∼1334–1342	+	+	+	Proteins, C–H deformation
1376	+			DNA, ring breathing modes of adenine
1399		+		Cytochrome c
1444	+	+	+	CH_2_ and CH_3_ deformations (antisymmetric methyl and methylene deformations, peptide side chains, phospholipids),—CH_2_ bending mode of proteins and lipids,—CH_2_CH_3_ deformation,—C–H vibration (proteins and lipids),—CH_2_ bending mode of DNA, CH_2_ deformation (thymine); C = C bending mode of guanine
1452	+	+		Cardiolipin
1578				Nucleic acid DNA modes: guanine, adenine ring breathing modes
1582		+		Cytochrome c
1635		+		Cytochrome c oxidized form
∼1660–1665	+	+	+	Proteins, Amide I/C=C vibrations; adenine, thymine, guanine, cytosine (ring breathing modes of the DNA/RNA bases)
∼2876–2881	+	+	+	Lipids and proteins, symmetric stretching of—CH_2_ and CH_3_
2928	+	+	+	Proteins, DNA, symmetric stretching of CH_3_
3057	+	+	+	C-H ring

**FIGURE 3:**
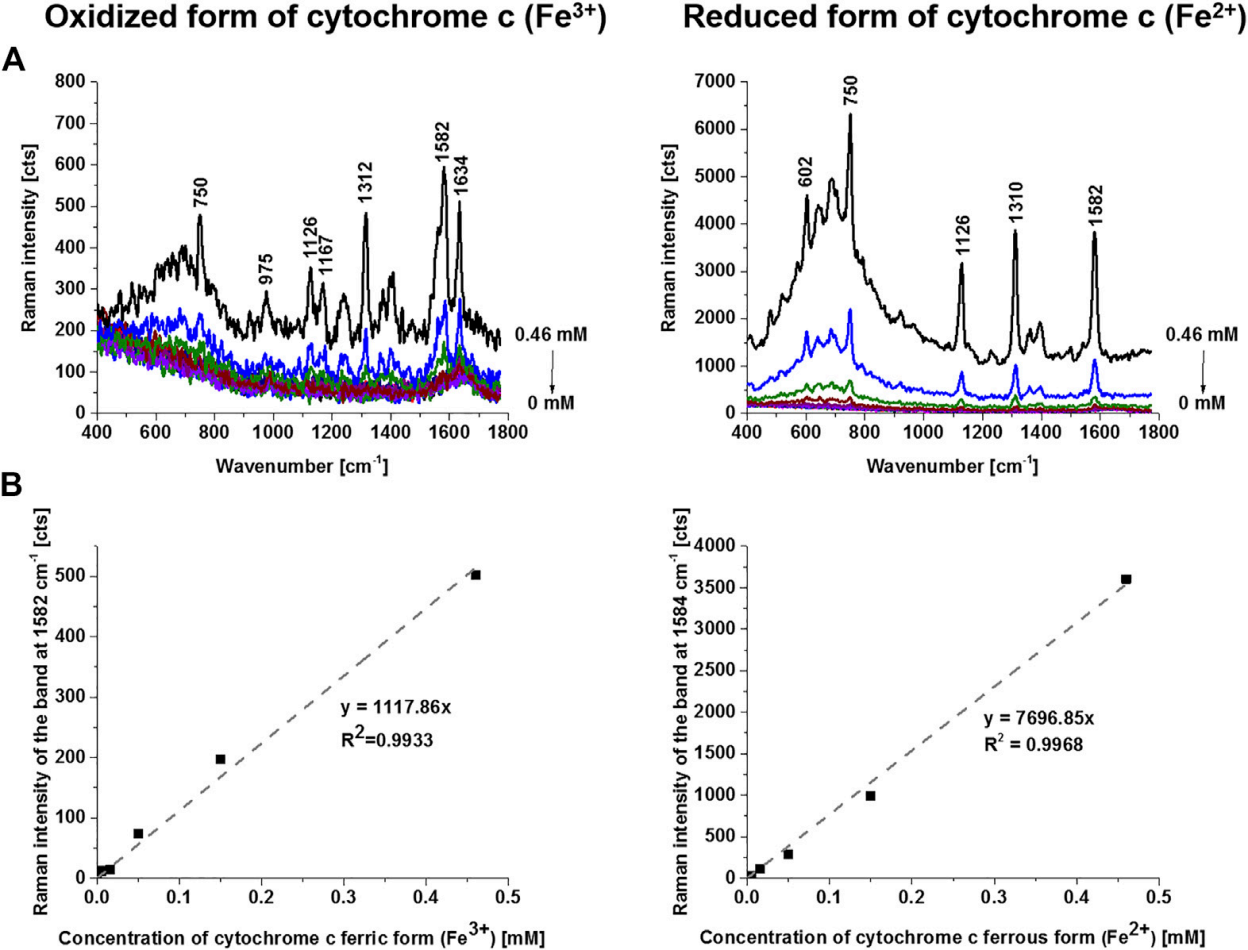
Raman spectra of oxidized (ferric form, Fe^3+^) and reduced (ferrous form, Fe^2+^) forms of cytochrome c as a function of concentration (PBS solutions (0–0.46 mM) **(A)**, Raman band intensity at 1582 cm^−1^ as a function of cytochrome c concentration **(B)**. Experimental conditions: excitation 532 nm, laser power 10 mW, integration time 0.5 s, 10 accumulations, 1 mm light path quartz cuvette. Reduction agent NaBH_4_ in tenfold excess.

The cytochrome c can exist in two forms: reduced (Fe^2+^) and oxidized (Fe^3+^). To estimate the concentration of cytochrome c for the oxidized and reduced forms we performed the reference curves for correlation between the Raman intensities of the oxidized and reduced forms of cytochrome c and their concentrations. Figure 3 shows the intensity of the Raman peak centered at 1582 cm^−1^ as a function of cytochrome c concentration for the oxidized and reduced forms measured at the same experimental conditions as used for the sperm cells. Figure 3 shows that both forms have the Raman band of the heme group vibrations at 1582 cm^−1^, but the Raman intensity of the reduced form is drastically higher as presented in Figure 3.

**FIGURE 4:**
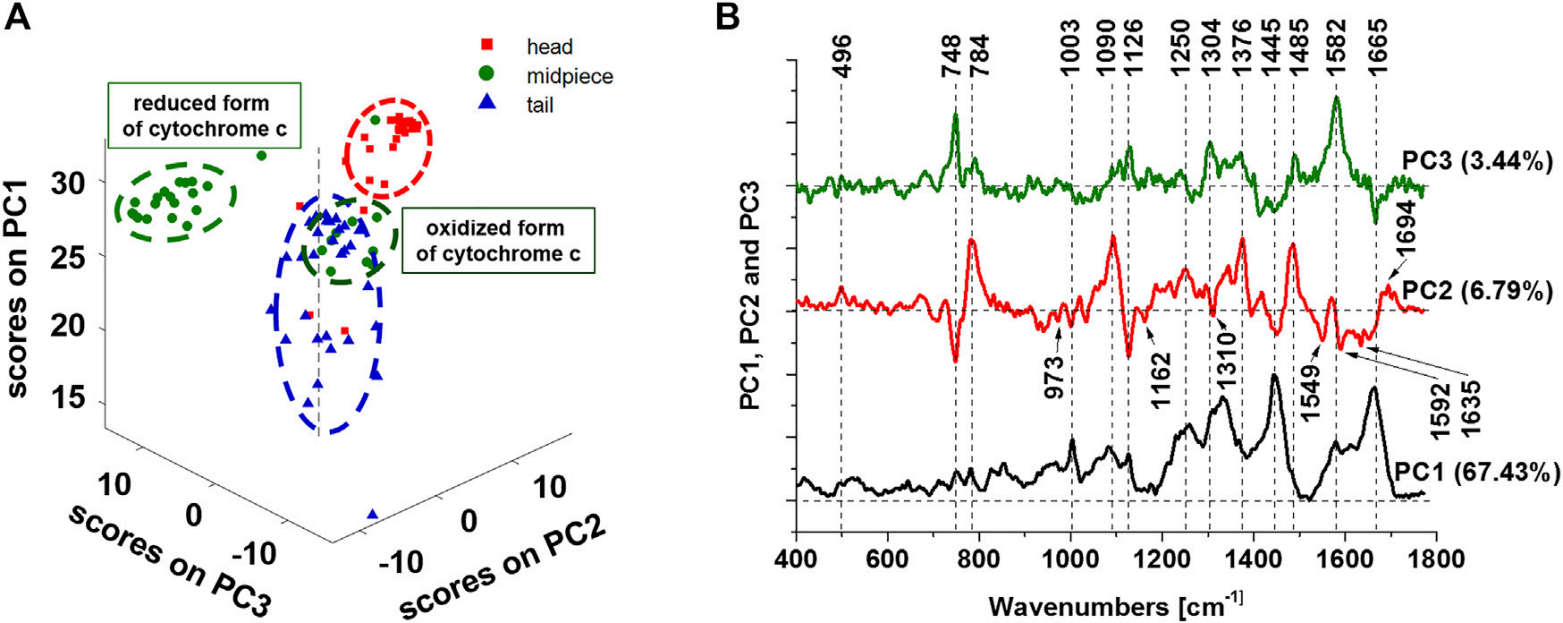
Principal components analysis of Raman data. The scatter plot of the score values of Raman spectra for the first, second and third principal components (PCs) from the head (red squares), midpiece (green circles) and tail (blue triangles) of sperm **(A)**. The loadings plots of PC1, PC2 and PC3 **(B)**. Data was acquired at 532 nm with 0.5 s integration time and 10 accumulations per single spectrum. The total number of 90 line scanned Raman spectra from the sperm cells were analyzed. Preprocessing PCA mode: SNV normalization.

Now we present principal components (PCA) analysis of all line scanned Raman data (number of Raman spectra of each line *n* = 10) presented in Figure 2B to visualize the vibrational features for a better understanding of biochemical hallmarks of the sperm regions (heads, midpiece and tail). The distribution of the scores on principal components 1, 2, and 3 presented in Figure 4 A clearly distinguishes the head, midpiece and tail regions of sperm cells. The loadings plots in Figure 4B helps to identify the chemical components of the sperm regions. The PCA results in Figure 4B confirm that the midpiece (green dots) is characterized by the redox-balanced two forms of cytochrome c. Positive PC3 values–Raman bands at 748, 1126, 1304, and 1582 cm^−1^ are characterized by a reduced form of cytochrome c, while the oxidized form exhibits negative values on PC2 at 748, 1126, 1310, and 1635 cm^−1^. Positive values on PC2 at 784, 1090 and 1376 cm^−1^ characterize DNA rich region (nucleus).

## 4 Discussion

It has been reported that mitochondria have a significant contribution to regulating the various physiological aspects of reproductive processes. Normally functioning mitochondria and intact mitochondrial membrane potential are a pre-requisite for normal sperm motility (Piomboni et al., 2012; Dutta et al., 2019; Ribeiro et al., 2021), hyperactivation (Dutta et al., 2019), capacitation (Jha et al., 2003; Bailey, 2010; Aitken, 2017; Dutta et al., 2019), acrosin activity (Breitbart, 2003), acrosome reaction (Breitbart, 2003; Gupta and Bhandari, 2011)and DNA integrity (Sakkas and Alvarez, 2010; Mallidis and Wistuba, 2018; Durairajanayagam et al., 2021; Podolak et al., 2022).

The results presented in Figure 2 demonstrate that sperm cells exhibit particular sensitivity to the redox status of cytochrome c in mitochondria. Our results show that cytochrome c is located in sperm mitochondria in the oxidized or reduced form of the heme group. The results from Figure 3 compared with those presented in Figure 2 provide the first direct evidence that the redox balance of sperm cells differs significantly for different cells. The sperm cell in Figure 3B (cell 1) represents cytochrome c in the oxidized form [cytochrome c (Fe^3+^)], for cells 2 and 3 the redox balance is significantly shifted to the reduced form [cytochrome c (Fe^2+^)]. The normal functioning of sperm cells requires cytochrome c to be in the oxidized form in the mitochondrial intermembrane space (Brown and Borutaite, 2008; Ow et al., 2008; Abramczyk et al., 2021; Abramczyk et al., 2022). This ensures that electron shuttling between the complex III, cytochrome c, and complex IV can occur leading to controlled effective oxidative phosphorylation (respiration) and ATP production. ATP production is needed to provide energy for most steps in spermatozoal maturation, motility, hyperactivation and fertilization. In contrast, for the sperm cells in Figure 3B (cell 2 and 3) with the reduced form (Fe^2+^) this process is blocked.

It is worth emphasizing that the band at 1444 cm^−1^ representing C-H vibrations of saturated lipids in the oxidized cytochrome c in Figure 2B (cell 1) is shifted to 1452 cm^−1^ for the reduced forms of cytochrome c (cells 2 and 3 I Figure 2B). The frequency at 1452 cm^−1^corresponds to cardiolipin, which plays an important role in the electron transport in the respiratory chain (Fikiet and Lednev, 2019).

Cardiolipin-bound cytochrome c, probably does not participate in electron shuttling of the respiratory chain (Unsay et al., 2013), and the reduced cytochrome cannot induce the caspase and apoptosis process (Abramczyk et al., 2022).

Therefore, Figures 2A,B (cell 1) represent the normal sperm cell. The signal at 1582 cm^−1^ for the normal sperm cells represents predominantly the oxidized form (Fe^3+^) of cytochrome c unbound to cardiolipin [low Raman intensity at 1582 cm^−1^ and lipids represented by the band at 1444 cm^−1^ uncoupled to cardiolipin (Figure 2B)].

We suggest that the quality of the sperm cells and their fertility depends on the redox balance of cytochrome c shifted to the oxidized form in cell mitochondria sperm cells. Thus, the normal functionality of sperm cells is assured by the oxidized form of cytochrome c localized in mitochondria being a part of the electron transport chain (ETC) resulting in normal effectiveness of respiration (oxidative phosphorylation), apoptosis, and normal ATP production. In contrast, Figure 2 (cell 2 and cell 3) represents the reduced form of cytochrome c, which defects sperm mitochondrial function and severely impairs the maintenance of energy production required for sperm motility and may be an underlying cause of infertility. We hypothesize that the redox balance effects also acrosome activity in producing hydrolytic enzymes that enable it to enter the egg cell and thus fertilize.

A greater understanding of sperm mitochondrial function and its correlation with sperm quality could provide further insights into their contribution to the assessment of the infertile male (Durairajanayagam et al., 2021).

## 5 Conclusion

It has been demonstrated that label-free Raman imaging provides biochemical characterization and functional status of specific organelles such as mitochondrion and nucleus in sperm cells. It was found that the most important structures of sperm such as the head consisting of the nucleus and acrosome, the midpiece representing mitochondria, and the tail characterized by the sperm axoneme surrounded by outer dense fiber and covered by the membrane could be clearly distinguished by Raman imaging. Our results show that cytochrome c, which is a key protein that is needed to maintain life (respiration) and cell death (apoptosis), is located in sperm mitochondria in the oxidized or reduced form of the heme group. The normal functioning of sperm cells requires cytochrome c in the redox balanced form: reduced and oxidized. The oxidized form of cytochrome c is localized in the mitochondrial intermembrane space and is a part of the electron transport chain. This ensures that electron shuttling between complex III, cytochrome c, and complex IV can occur leading to controlled effective oxidative phosphorylation and ATP production. We showed that Raman imaging may be applied for rapid fertility testing from the redox status of mitochondria in sperm cells. Further experiments are needed to provide the final output in the form of protocols for ‘real’ sample examination for clinical trials, but it will be the next stage in our research and cooperation with the medical centers.

## Data Availability

The original contributions presented in the study are included in the article/supplementary material, further inquiries can be directed to the corresponding authors.
